# On the Development of an Effective Method to Produce Conductive PCL Film

**DOI:** 10.3390/nano11061385

**Published:** 2021-05-24

**Authors:** Giacomo Damonte, Alberto Vallin, Alberto Fina, Orietta Monticelli

**Affiliations:** 1 Dipartimento di Chimica e Chimica Industriale, Università degli studi di Genova, Via Dodecaneso 31, 16146 Genoa, Italy; giacomo.damonte@edu.unige.it (G.D.); berto.vallin@gmail.com (A.V.); 2 Dipartimento di Scienza Applicata e Tecnologia, Politecnico di Torino-sede di Alessandria, Viale Teresa Michel, 5, 15121 Alessandria, Italy; alberto.fina@polito.it

**Keywords:** PCL, nanocomposites, graphite nanoplates, compatibilization, melt blending, electrical conductivity

## Abstract

The aim of this work was to develop an effective approach to improve the graphite dispersion and, consequently, the electrical conductivity of nanocomposites based on polycaprolactone (PCL) and graphite nanoplates (GNP). With this aim, a polymeric additive was designed to be compatible with the polymer matrix and capable of interacting with the graphite layers. Indeed, the compound consists of a low molecular mass PCL ending with a pyrene group (Pyr-PCL). The exploitation of such a molecule is expected to promote from one side specific interactions of the pyrene terminal group with the surface of graphite layers and from the other to guarantee the compatibility with PCL, having a chain with the same nature as the matrix. The features of the nanocomposites prepared by directly blending PCL with GNP were compared with those of the same systems also containing the additive. Moreover, a neat mixture, based on PCL and PCL-Pyr, was prepared and characterized. The specific interactions between the ad hoc synthesized compound and graphite were verified by UV measurements, while SEM characterization demonstrated a finer dispersion of GNP in the samples containing Pyr-PCL. GNP nucleating effect, proved by the increase in the crystallization temperature, was observed in all the samples containing the nanofiller. Moreover, a significant improvement of the electrical conductivity was found in the systems based on the pyrenyl terminated PCL. This peculiar and interesting phenomenon was related to the optimized nanofiller dispersion and to the ameliorated compatibility with the polymer matrix.

## 1. Introduction

The combination of graphene and graphene-related materials (GRM) [1] with biopolymers represents an appealing and effective approach to enlarge the exploitation of such systems, which represent a valid alternative to polymers from fossil sources [2,3,4]. Indeed, on one hand, the addition of the above nanoparticles could potentially improve the features of the polymer matrix and disclose novel properties and on the other, the low environmental impact of both the components make the resulting composite/nanocomposites “green”. Clearly, in the development of these materials it is necessary to consider the exploitation of preparation methods capable of facilitating the nanoparticle dispersion, easily applicable and sustainable. With regard to the latter aspect, the combination of GRM with the biopolymers was generally carried out by using solution-mixing approaches [2] or via melt blending [2,3], which is highly recommended for industrial viability. The layered carbon filler dispersion, such as graphite nanoplates (GNP), which is the object of the present work may be challenging and depends on the affinity towards the polymer matrix. Partial oxidation of graphite using strong acids and oxidating agents was widely used to prepare graphite oxide or graphene oxide (GO) [5,6], which may enhance the affinity towards polar polymers. In the case of the investigated biopolymer, namely poly(ε-caprolactone) (PCL), it was mainly combined with GO by applying the solution blending [7] or the in-situ polymerization method [8,9]. Thanks to the filler functionalities, by using the latter approach, a direct grafting of the macromolecular chains onto the surface of GO was achieved, allowing a strong interfacial interaction between GO and the PCL matrix. Moreover, studies on the crystallization of PCL/GO nanocomposites, prepared by using the in-situ polymerization method, showed that the crystallization temperature of the polymer was significantly enhanced with respect to the neat polymer, without affecting the crystalline structure [8]. As demonstrated by Wang et al. [9], the direct PCL grafting improved not only graphene dispersion in the polymer matrix, but also the mechanical properties of the resultant composites. Electrospinning is another approach exploited to combine GO with PCL: in a pioneering work, describing the preparation of PCL/GO nanocomposites by applying the above method, a significant increase in the mechanical strength by the incorporation of GO was found, which phenomenon was directly related to the changes in the fiber morphology [10]. Furthermore, the composite nanofiber mats turned out to be suitable scaffolds, showing high bioactivity [11,12]. Despite the potentialities of such materials, it is worth underlining that in order to restore high electrical and thermal conductivity, reduction of GO has to be accomplished, which requires the use of strong chemical reducing agents and/or extremely high temperature [13]. As such, thermally reduced graphene oxide (TRGO) was incorporated in a PCL matrix via the conventional solution casting method [7]. The developed nanocomposites were characterized by a fine dispersion of TRGO throughout the PCL matrix, which lead to a significant improvement in the storage modulus. The combination of GRM with PCL was also used in the development of blends with polylactic acid (PLA) [14,15,16,17]. Indeed, nanoparticles were found to be capable of decreasing the surface tension between the two polymers, thus acting as a compatibilizer. In particular, using GNP, the electron microscopy measurements indicated a predominant localization of nanoplates in the PCL phase [14]. It is worth underlining that GRM dispersion represents a key issue for the above systems, as it is necessary to reach a fine distribution to transfer the properties of the nanofiller to the polymer matrix. In this respect, while the oxidation of graphite and possible subsequent organic functionalization may allow better interaction with the polymer matrix, enabling easier nanofiller dispersion, the chemical modification of GRM introduces disruptions of the sp^2^ structure, thus affecting their physical properties, particularly in terms of electrical and thermal conductivity. An alternative approach to guarantee strong interactions between non functionalized GRM is the modification of the chemical structure of the macromolecules to promote non-covalent bonding with graphitic surfaces. As such, taking into account the specific interactions that occur between pyrene molecules and the surface of the graphite lamellae [18,19], polymers bearing these functionalities were developed and used for the preparation of composite systems [20,21,22]. In particular, considering biopolymers, in a recent work of ours we reported on the development of nanocomposites based on poly(l-lactide) (PLLA) by synthesizing initiators, constituted by a pyrene end group and a poly(d-lactide) (PDLA) chain, capable of interacting with the surface of GNP layers as well as forming stereoblocks during the ring opening polymerization of l-lactide [22]. Clearly, the application of the pyrene-based functionalization to other biopolymers by using environmentally friendly routes with sustainable and scalable processing to obtain graphite nanocomposites is of great interest. Within this scenario, this work represents the first report on PCL-based pyrene (Pyr-PCL) systems to be used as additives in the preparation of PCL/GNP nanocomposites. In order to maintain the sustainability of the whole process, the synthesis of Pyr-PCL was carried out without using solvents and the blends were prepared via a simple and industrially viable melt blending procedure. The developed materials were characterized by using DSC, TGA, FE-SEM and electrical conductivity measurements.

## 2. Materials and Methods

### 2.1. Materials

From Sigma Aldrich^®^ (Milan, Italy), ε-caprolactone (purity ≥ 97%), dimethylformamide (DMF) (purity ≥ 99%), 1-pyrenebutanol (purity ≥ 99%), 1-dodecanol (purity ≥ 99%), tin octanoate (Sn(Oct)_2_) (purity ≥ 96%), toluene (anhydrous, purity 99.7%), dichloromethane (stabilized with 0.002% 2-methyl-2-butene), and methanol (99.9%) were purchased. GNP was provided by Avanzare Innovación Tecnólogica (Navarrete (La Rioja), Spain), prepared according to a previously reported procedure [23]. Commercial PCL CAPA^®^ 6500 (M_w_ = 50,000 g/mol) was purchased from Perstorp (Malmö, Sweden). The ε-caprolactone was purified prior to use by vacuum distillation over CaH_2_. All the other reagents were of analytical grade and used without purification.

### 2.2. Synthesis of Pyr-PCL and Dod-PCL

The synthesis of 1-pyrenyl terminated PCL (referred to as Pyr-PCL) was carried out using the ring opening polymerization (ROP) reaction in bulk, a method widely reported in the literature for the preparation of polycaprolactone [24,25,26]. Briefly, 3.650 g (31.978 mmol) of ε-caprolactone was placed in a 50 mL round bottom flask equipped with a magnetic stirrer under argon atmosphere, then 0.500 g (1.822 mmol) of 1-pyrenebutanol was added and the system was slowly heated to 80 °C and stirred until complete dissolution of the initiator. The polymerization reaction was started by increasing the temperature to 120 °C followed by the addition of the catalyst, namely 26 µL of a freshly prepared 100 mg/mL solution (6.417 µmol) of tin octanoate (Sn(Oct)_2_) in anhydrous toluene (ratio [ε-CL]/[Sn(Oct)_2_] = 5000). The reaction mixture was maintained at 120 °C for 24 h under stirring. After cooling, the warm crude product was dissolved in 2 mL of CH_2_Cl_2_ and precipitated by adding the viscous solution obtained dropwise into 200 mL of cold methanol under slow stirring. After precipitation, the product was filtered on a Buchner funnel, washed with small amounts of ice cold methanol and then dried at 40 °C under vacuum for 72 h. 1-dodecyl terminated PCL (referred to as Dod-PCL) was synthesized by adjusting the initiator amount, namely 1-dodecanol, to obtain a polymer characterized by the same molecular mass as that of Pyr-PCL. The synthesis of Dod-PCL, with a theoretical M_n_ of 2000 g/mol, was performed using the same method applied for the preparation of the pyrenyl terminated PCL. In this case, the ROP was performed using 3.260 g (28.561 mmol) of ε-caprolactone and 0.304 g (1.631 mmol) of 1-Dodecanol. The polymerization reaction was started by adding 23 µL of tin octanoate solution.

### 2.3. Preparation PCL/GNP Nanocomposites

Neat mixture (referred to as PCL/Pyr-PCL) was prepared by mixing a commercial PCL (referred to as PCL) with the ad hoc synthesized 1-pyrenyl terminated PCL, using a ratio PCL/Pyr-PCL of 80/20. Before blending at 120 °C for 30 min, the polymers were dried overnight at 40 °C under vacuum. The mixture was prepared by using a laboratory internal mixer equipped with a mechanical stirrer, type RZR1 (Heidolph Instruments GmbH & Co, Schwabach, Germany), which was connected to a vacuum line and evacuated for 30 min at room temperature, followed by purging with Argon for 30 min (the above operations were repeated at least three times, to be sure to avoid humidity coming in contact with the reagents). The reactor was heated at 120 °C and stirred for 5 min at 160 rpm under inert atmosphere. GNP at two different concentrations (1 and 2 wt.-%) was added to the 80/20 blends to prepare composite systems, which were assigned names indicating the quantity of the mixed graphite (as an example PCL/Pyr-PCL/G1 indicates a sample based on a ratio PCL/Pyr-PCL 80/20 and a percentage of GNP of 1 wt.-%). Before mixing the nanofiller with the polymer matrix, it was pre-dispersed into a round bottom flask with toluene using a sonicating bath at 40 kHz for 60 min. Successively the solvent was removed, using a rotavapor, and the sonicated material was collected. The same conditions were applied for the composites, which were prepared by mixing the polymers and graphite. The neat mixture and the composites were dried under vacuum at 40 °C for 72 h. In order to evidence the effect of the pyrenyl-based additive on the composite characteristics, samples were prepared by adding the graphite directly to the neat commercial PCL, by using the same equipment and conditions previously described.

### 2.4. Polymers Characterization

^1^H-NMR measurements were recorded on a Varian “Mercury 300”(Palo_Alto, CA, USA)operating at a frequency of 300 MHz. All the samples were dissolved in CDCl_3_ in 10 mm NMR tubes at room temperature. The sample concentration used was 30 mg/mL. The thermal properties were measured using a Mettler Toledo “DSC1 STARe System” (Milan, Italy) differential scanning calorimeter (DSC) in the temperature range from −100 to 150 °C under a nitrogen flow of 20 mL/min and employing 40 µL aluminum crucibles with pin. The heating and cooling rates were +10 (heating) and −10 (cooling) °C/min. The thermogravimetric analysis (TGA) was performed using a Mettler Toledo “DSC1 STARe System” in the temperature range from 30 to 800 °C, at a heating rate of 10 °C/min under a nitrogen atmosphere. The FT–IR spectra were acquired with a Bruker “Vertex 70” operating in ATR mode with a diamond crystal, from 400 to 4000 cm^−1^. UV–Vis measurement were performed using a Shimadzu “UV-1800” (Milan, Italy) spectrometer equipped with short path quartz cells (0.2 cm, slit: 1 nm, scan speed: very slow). In particular, in order to evaluate the interaction between Pyr-terminated PCL and graphite, 0.1 mg of Pyr-PCL was dissolved into a test tube with 2 mL of DMF, then different amounts of GNP (0, 0.5, 1, 2, and 5 mg) were added. The five samples were sonicated using a sonicating bath (40 kHz, maximum power) for one hour. After sonication, the dispersed samples were left to sedimentate at room temperature for one week. The solution surnatant was collected and analyzed spectroscopically from 270 to 1100 nm to show the content of pyrenic units.

Surface conductivity tests were performed at room temperature by applying a picoammeter (Keithley Instruments, Solon, OH, USA) and by using films of 12 × 10 mm (with a thickness of ca. 0.2 mm). Two rectangles of conductive tape (10 × 1 mm, 3M electrically conductive “ECATT” adhesive tape 9707), spaced 10 mm apart, were deposited on the films surface in order to form the electrical contact. For the measurements, a ddp. of 150 V cc. was applied.

## 3. Results and Discussion

### 3.1. Synthesis and Characterization of Pyr-PCL

#### 3.1.1. H-NMR Analysis

**Figure 1:**
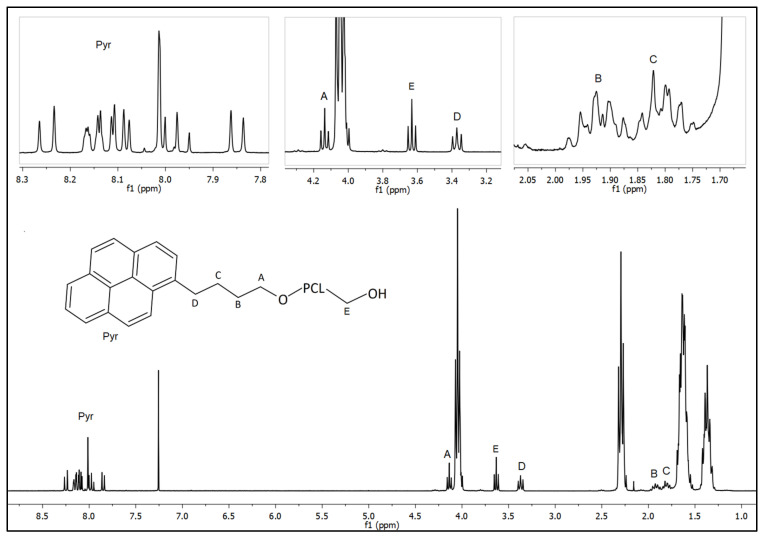
^1^H-NMR spectra of Pyr-PCL in CDCl_3_.

This work has been primarily focused on the fine-tuning of the synthesis of a pyrenyl terminated PCL (Pyr-PCL), prepared by applying the ROP of ε-caprolactone and by using 1-pyrenebutanol as initiator. In order to evidence the influence of the pyrenyl group on the specific features of the above polymer, a dodecyl terminated PCL (Dod-PCL) was also synthesized, adjusting the initiator/monomer ratio to obtain two polymers characterized by the same molar mass, namely 2000 g/mol, but different end groups. It is worth underlining that Dod-PCL was not applied in the nanocomposite preparation. In order to calculate the molar mass (M_nNMR_), as well as to confirm the synthesized polymer structures, ^1^H-NMR measurements were performed. Figure 1 shows the ^1^H-NMR spectrum of Pyr-PCL. Indeed, M_nNMR_ was calculated using the ratio between the intensities of the triplet peaks around 2.29 ppm and (E) 3.63 ppm. The value found for both the polymers was ca. 2000 g/mol, which is in accordance with the theoretical number for the average molecular weight, thus demonstrating the fine control of the polymerization reaction. It was also possible to recognize the other signals correlated to the methylenic couples of protons in PCL repetition units at 4.05 (t), 1.64 (m), 1.38 (m). The Pyr-PCL sample also displayed a distinct pattern around 8 ppm (Pyr) given by the aromatic pyrene protons, along with the presence of four butylic chain signals (A) 4.14 (t), (D) 3.37 (t), (B) 1.92 (quint), and (C) 1.80 (quint) ppm. The chemical shift value of the signal A confirmed that all the initiators reacted to form covalent links with PCL chains.

#### 3.1.2. FT-IR Analysis

**Figure 2:**
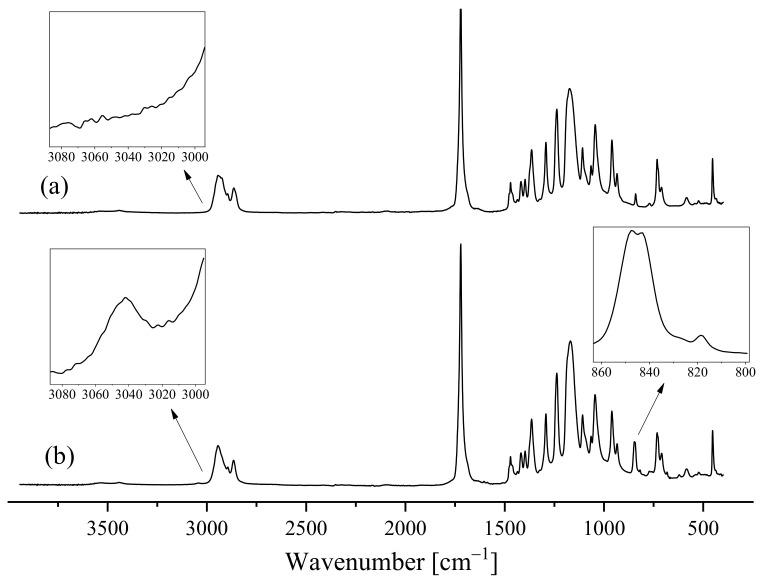
FT-IR spectra of Dod-PCL (**a**) and Pyr-PCL (**b**).

In order to further corroborate the chemical structure of the two polymers, FTIR measurements were performed. The comparison of the Pyr-PCL and Dod-PCL spectra is reported in Figure 2. For both samples, it is possible to recognize the signals typical of PCL chains [27] at 3400 cm^−1^ (O–H hydroxyl moiety stretching); 2950 cm^−1^ and 2870 cm^−1^ (symmetrical/unsymmetrical stretching Csp^3^-H bonds in methylenic unit); 1728 cm^−1^ (carbonyl stretching); 1296 cm^−1^ (C–O and C–C stretching); and at 1241 cm^−1^ and 1170 cm^−1^ (unsimmetrical/simmetrical C–O–C stretching). In the pyrenyl terminated sample novel bands appear at 3047 cm^−1^ (Csp^2^-H stretching); 1600 cm^−1^ (breathing of the pyrenic unit); 845 cm^−1^, 820 cm^−1^, 684 cm^−1,^ and 622 cm^−1^ related to the complex vibrational motions of pyrene.

#### 3.1.3. DSC Measurements

**Figure 3:**
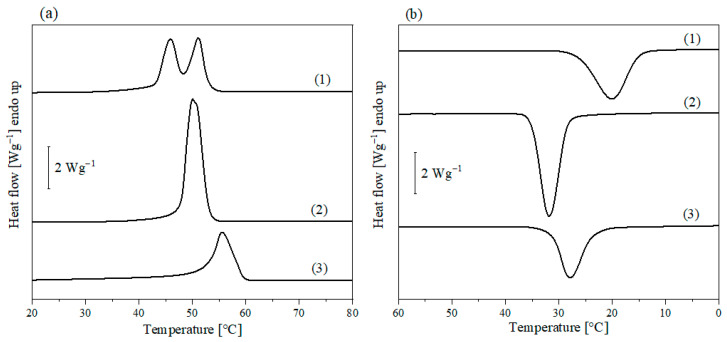
DSC thermograms of linear PCL, second heating (**a**) and cooling (**b**): (1) Pyr-PCL, (2) Dod-PCL, (3) PCL.

In order to evidence the effect of the functionalization and molecular mass on the polymer thermal properties, Dod-PCL, Pyr-PCL and a commercial PCL were analyzed by means of DSC. Figure 3 shows the cooling and heating traces for the above three samples, while the thermal data are summarized in Table 1. Differences in the thermal behavior can be noticed by comparing the DSC traces of two polymers which hold similar molecular mass, but different end groups. Indeed, while the melting temperature (T_m_) is similar for both Dod-PCL and Pyr-PCL, ca. 50 °C (Figure 3a), Pyr-PCL holds a double melting peak. This phenomenon is related to the crystal lamellae thickness distribution produced during crystallization [28,29], which is possibly affected by the presence of bulky pyrene moieties as chain ends on relatively short PCL chains. It is worth noting that the commercial PCL has a higher melting temperature (ca. 56 °C), reflecting its high molar mass [30]. Furthermore, Pyr-PCL shows a significantly lower crystallization temperature (T_c_) and crystallinity (χ_c_) with compared with Dod-PCL. This result can be related to the steric hindrance of the pyrenyl group, which may affect the polymer structuring. The specific effect of the initiator on the crystallization behavior of PCL has also been reported in the literature for other kind of systems [30,31]. In the case of the commercial PCL, the lower crystallinity and T_c_ can be ascribed to the higher molecular mass with respect to both Pyr-PCL and Dod-PCL.

#### 3.1.4. TGA Measurements

**Figure 4:**
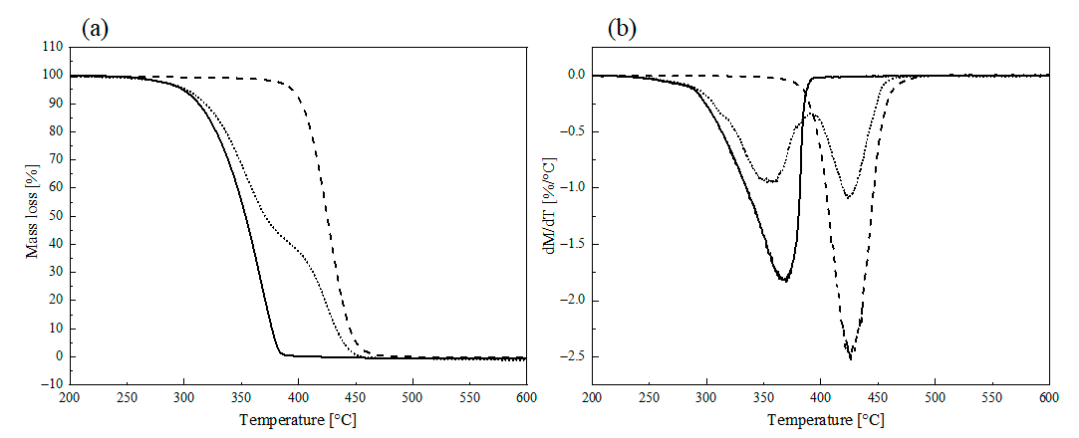
TGA (**a**) and DTG thermogram (**b**): Pyr-PCL (continuous line), Dod-PCL (dotted), PCL (dashed).

The TGA curves of Pyr-PCL, Dod-PCL and PCL are reported in Figure 4. While Dod-PCL shows two degradation steps, the TGA profiles of Pyr-PCL and PCL are characterized by only one decomposition step. In order to elucidate this finding, it is necessary to consider the specific degradation behavior of polycaprolactone. As reported in the literature [32,33,34], the decomposition of this polymer follows two concurring mechanisms, namely unzipping, which requires a nucleophilic terminal chain group such as hydroxyl functionalities, and β-elimination, which needs at least a hydrogen atom onto the β-carbon in the alcoholic side of the ester moiety. The commercial PCL decomposes in one weight loss step (400–450 °C), suggesting the effect of terminal OH groups is negligible, in accordance with its high molecular mass. On the other hand, in the case of the low molecular mass polymers, the high concentration of hydroxyl groups is likely to promote the unzipping mechanism, leading to an anticipation of the weight loss onset. However, while Pyr-PCL is completely volatilized before 400 °C is reached, Dod-PCL also displays a second weight loss step at higher temperature (ca. 425 °C), which may be related to the β-elimination, apparently hindered for Pyr-PCL because of the pyrenyl end group.

### 3.2. Study of the Graphite Dispersion

**Figure 5:**
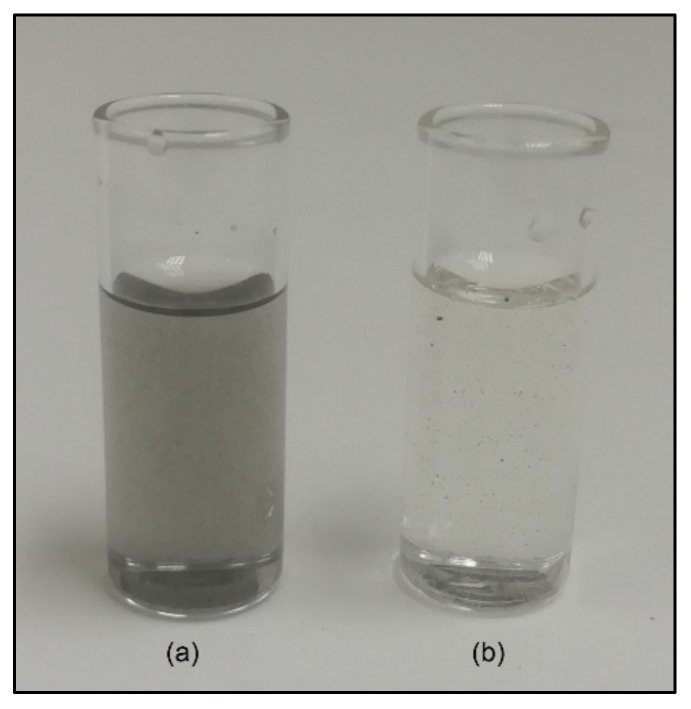
Pyr-PCL DMF solution + GNP (**a**), neat DMF + GNP (**b**); both after bath sonication for 1 h and 24 h of sedimentation. Quantity used: 3 mL of DMF, 50 mg of polymer and 5 mg of graphite.

The capacity of the pyrenyl terminated polymer to disperse and stabilize GNP in a liquid medium was primarily evaluated by comparing two dispersions in DMF, one containing only GNP and the other GNP and Pyr-PCL. Figure 5 shows the photos of the two dispersions after a sonication and centrifugation treatment. Indeed, the neat DMF was not capable of stabilizing the GNP as evidenced by the transparency of the liquid. Moreover, in the mild sonication conditions used, GNP is not expected to exfoliate in DMF and leads to a poorly stable suspension which fully precipitates after several hours.

**Figure 6:**
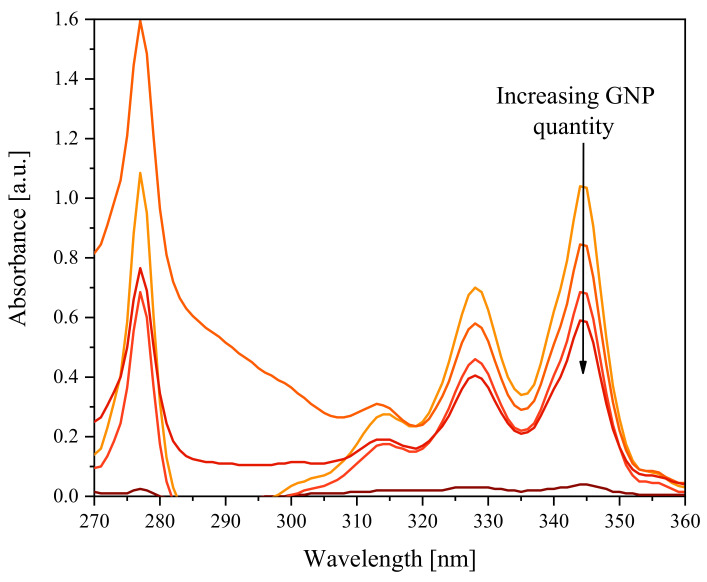
UV-Vis spectra of Pyr-PCL DMF solution surnatant with different amounts of GNP, after sonication and 7 days of sedimentation: neat, 0.5 mg, 1 mg, 2 mg, 5 mg from orange to brown.

Conversely, the suspension containing Pyr-PCL, appears slightly grey even after 24 h, thus demonstrating that the polymer promoted stabilization of the GNP suspension. A more detailed analysis of the specific interactions between graphite and the pyrenyl terminated polymer was obtained by UV measurements. Figure 6 shows the UV spectra of the surnatants, which were prepared by adding Pyr-PCL and different amounts of graphite to DMF. In the acquired spectra it is possible to observe, in the range 310–350 nm, strong adsorption bands characteristic of the pyrenic unit [35], whose intensity significantly decreased by increasing the graphite amount. This finding suggests the adsorption of Pyr-PCL onto the surface of graphite flakes, likely due to the pyrenic unit π-stacking interactions [36]. Another interesting piece of information comes from the shoulder of the band at 280 nm, which can be ascribed to the absorption produced by the dispersed graphite flakes. This signal started to be noticeable in the sample containing 0.5 mg of graphite. This phenomenon demonstrated that Pyr-PCL promotes the dispersion of graphite when the ratio of Pyr-PCL/graphite reaches a critical value, which allows the adsorption of the molecule on the surface of the flakes as well as maintaining the GNP dispersed in DMF, thus avoiding its coalescence. The proven adsorbance of Pyr-PCL onto GNP suggests the possibility to exploit this as a compatibilizer in PCL nanocomposites [22].

### 3.3. Preparation and Characterization of PCL/GNP Nanocomposites

**Figure 7:**
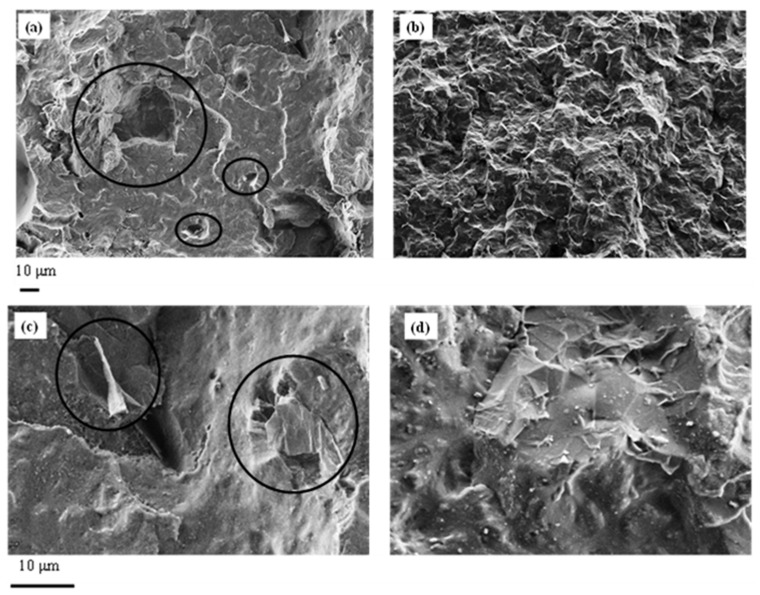
FE-SEM micrographs at different magnifications of: (**a**,**c**) PCL/G1 and (**b**,**d**) PCL/Pyr-PCL/G1.

FE-SEM micrographs of the samples PCL/G1 and PCL/Pyr-PCL/G1 are given in Figure 7. The former composite, prepared by adding directly GNP to the PCL, was characterized by an inhomogeneous dispersion of the filler and by the presence of several voids, formed by the GNP removal during the fracture (see insert in the Figure 7a).

This phenomenon can be related to the poor adhesion between the polymer matrix and the filler. Conversely, a more homogeneous distribution of GNP was found in the Pyr-PCL-based sample, whose micrograph evidenced the strong adhesion of the GNP with PCL (see insert in the Figure 7b). This result clearly demonstrates that the previously mentioned interactions, occurring between the pyrenyl terminated polymer and graphite, also promote the filler dispersion in the molten system as well as the adhesion of the PCL to the GNP. It is worth underlining that very similar morphologies were also found for the composites based on 2 wt.-% of GNP. In Table 1, thermal data of the neat samples (Pyr-PCL, Dod-PCL and PCL) are compared with the system based on PCL and Pyr-PCL (PCL/Pyr-PCL) as well as with those containing GNP, prepared by directly adding the nanoplates to the PCL or to the blended PCL/Pyr-PCL. Neat PCL/Pyr-PCL exhibited the same crystallization temperature (T_c_) as PCL but was characterized by a higher crystallinity (χ_c_). This result can be related to the presence of the low molecular mass polymer, which might reduce the viscosity of the molten system, thus helping the polymer structuring. For all the composite samples, a significant increase in T_c_ and a slight enhancement of the crystallization with respect to PCL were observed. These phenomena, already reported in the literature [37], indicate that GNP acts as a nucleating agent for the PCL structuring. Concerning the thermal decomposition of the prepared compounds, T_onset_ of the systems based on Pyr-PCL showed a slight decrease with respect to the neat PCL, which phenomenon might be accounted for by the presence of the low molecular mass polymer. Nevertheless, it is worth underlining that T_max_ was found to be constant for all the analyzed systems.

The effect of GNP on the electrical properties of the prepared systems was evaluated by comparing the conductivity (σ) of the neat samples with those of the composites (Table 1). While the polymer matrices are electrically insulating with σ in the range of 10^−11^ S·m^−1^, the addition of GNP to PCL led to an increase in the electrical conductivity. In the case of the samples prepared by directly adding GNP to PCL, σ turned out to be similar, the conductivity values only slightly decreasing by increasing the graphite content, passing from 1.7·10^−10^ to 8.3·10^−11^ in the samples containing 1 and 2 wt.-% of GNP, respectively.

On the other hand, samples based on Pyr-PCL showed a much higher σ, thus confirming better dispersion of GNP. For these nanocomposites, the conductivity was found to increase by increasing the amount of GNP, reaching a value of 5·10^−2^ for the sample PCL/Pyr-PCL/G2. The different behavior found in the nanocomposites based on the neat PCL with respect to those containing the additive, can be explained by taking into account that Pyr-PCL renders GNP more compatible with the polymer matrix, allowing the addition of a greater quantity of the nanofiller before reaching its coalescence, a phenomenon which generally leads to a reduction in the material final properties.

**Figure 8:**
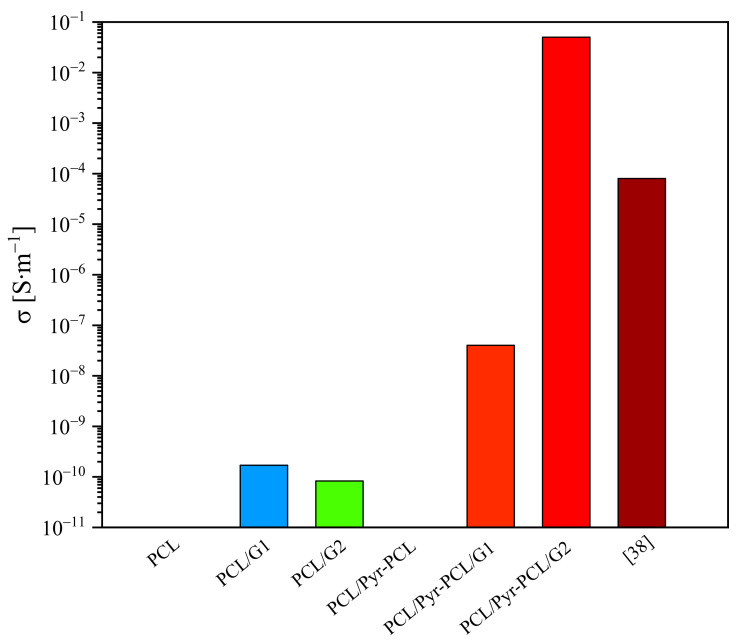
Surface conductivity of a commercial PCL, of our developed samples and of a PCL-based system reported in the literature.

While conductive systems based on PCL were generally prepared by using reduced graphene oxide (rGO) and by applying the solution mixing method [38,39], in this work, we demonstrated an alternative approach. Indeed, the exploitation of the ad-hoc synthesized additive on one hand avoids the use of oxidized forms of graphite, and on the other, allows preparation via melt blending, which is more easily scalable and environmental friendly compared to solution processing. Moreover, the conductivity, which was 10^−5^ in the case of a system reported in the literature [38], based on PCL and containing comparable amounts of graphite, turned out to be lower than the values found in our composites based on Pyr-PCL (Figure 8). These relevant results corroborate the active role of the synthesized pyrenyl terminated polymer in the graphite dispersion and consequently in the formation of an effective percolative network.

## 4. Conclusions

A novel polymer additive, consisting of a low molecular mass polycaprolactone ending with a pyrene group (Pyr-PCL), to be applied in the preparation of composites based on a commercial PCL and graphite nanoplates, was developed. The structure of the polymer, synthesized by applying the ROP of ε-caprolactone and by using 1-pyrenebutanol as initiator, was confirmed by ^1^H-NMR and FT-IR analysis. The comparison of the thermal behavior of Pyr-PCL with that of a dodecyl terminated PCL, characterized by the same molecular mass, evidenced that the pyrenyl group limits the polymer crystallization. The specific interactions, which occur between the pyrenyl terminated PCL and graphite flakes, as confirmed by UV measurements, turned out to affect the GNP dispersion in the nanocomposites. Indeed, with respect to the systems prepared by directly adding GNP to the molten polymer, the addition of Pyr-PCL allowed to obtain a finer dispersion and stronger adhesion of GNP to the polymer matrix. Pyr-PCL-compatibilized nanocomposites exhibited better electrical conductivity than that reported in the literature for other PCL/graphite systems. This was obtained by the use of poorly oxidized forms of graphite, by the design of Pyr-PCL as a compabilizer, and by exploiting an easily scalable melt blending process, opening up the possibility to use this biopolymer in novel and attractive applications.

## Figures and Tables

**Table 1:** DSC, TGA and surface conductivity measurement results.

Sample	ΔH_c_ [J/g]	T_c_ [°C]	ΔH_m_ [J/g]	T_m_ [°C]	χ_c_ [%]	T_onset 5%_ [°C]	T_Vmax1_ [°C]	T_Vmax2_ [°C]	σ [S·m^−1^]
Pyr-PCL	−79	20	83	51	60	298	367	-	-
Dod-PCL	−99	32	102	50	73	301	352	425	-
PCL	−63	28	72	56	51	394	-	426	<10^−11^
PCL/G1	−64	36	78	57	56	396	-	426	1.7·10^−10^
PCL/G2	−59	37	69	57	51	395	-	426	8.3·10^−11^
PCL/Pyr-PCL	−74	28	82	56	59	378	-	427	<10^−11^
PCL/Pyr-PCL/G1	−70	36	78	56	57	375	-	427	4.0·10^−8^
PCL/Pyr-PCL/G2	−79	38	87	56	64	376	-	429	5.0·10^−2^

ΔH_c_ = crystallization enthalpy, T_c_ = crystallization temperature, ΔH_m_ = melting enthalpy, T_m_ = melting temperature, χ_c_ = crystallinity percentage, T_onset 5%_ = temperature of 5% mass loss, T_Vmax1_ = temperature of the maximum degradation rate (first step), T_Vmax2_ = temperature of the maximum degradation rate (second step), σ = specific conductance.
